# Khaled Hosseini's women as modern archetypes: A study of obedient, resistant and empowered Afghan women

**DOI:** 10.3389/fsoc.2022.1041435

**Published:** 2022-11-30

**Authors:** Muhammad Imran, Sayed M. Ismail

**Affiliations:** ^1^Education Research Lab, Prince Sultan University, Riyadh, Saudi Arabia; ^2^University of Sahiwal, Sahiwal, Punjab, Pakistan; ^3^College of Sciences and Humanities, Prince Sattam Bin Abdulaziz University, Al-Kharj, Saudi Arabia

**Keywords:** Khaled Hosseini, Afghan women, patriarchal hierarchies, feminist insurgence, women's emancipation

## Abstract

This article focuses on Khaled Hosseini's use of the analogy of women as obedient, resistant, and empowered as modern archetypes who learn of the gendered oppression that works through their bodies. Khaled Hosseini, through his writings, lends voice and offers moral encouragement to women by crafting resistant, rebellious, empowered, and strong female characters. With theoretical support from Johnson's *Patriarchal Terrorism* and Spivak's *Can the subaltern speak*, this article traces how Hosseini interrogates the patriarchal hierarchies that encompass women's identity in Afghanistan. Women's sufferings correlate with the country's overall circumstances, particularly during and after the war, on the terror regime. Therefore, women's endurance and Afghanistan's endurance amid hostile and oppressive circumstances become equally imperative for Hosseini's works. This article finds that Hosseini correlates Afghan women's issues like tradition and modernity, women and Islam, mother and daughter relationship, resistance and rebellion, and their quest for change and empowerment with the war on terror, foreign invasions, and the rule of the Taliban in Afghanistan.

## Introduction

Middle Eastern women, especially Afghanistani women, are usually misunderstood to be weak, oppressed, and downtrodden subjects. Beyond the stereotyped portrayal of Afghanistani women through the Western media as submissive and in need of empowerment and liberation, people outside these countries do not know the journey traversed by these women from bondage to liberation as well as from awakening to determination (Imran, 2019). However, much of the challenge is curtailed by obnoxious incidents and stories about female early marriages, spousal abuse, violence, and patriarchal control over women's social, economic, and personal aspects of life. Moreover, the stereotyped portrayal of Afghan women covered from head to toe in *burqa*^1^ presents an offensive depiction of the Afghan society. Because of such a negative portrayal of women in media discourses, people outside Afghanistan usually rely on these media reports and build their opinions about women and their situation in Afghan culture.

The stereotyped depiction of Afghanistani women through media discourses within and outside Afghanistan often leads toward drawing rapid conclusions and a negative portrayal of the culture and society in Afghanistan. However, some international Afghan writers, like Asne Seierstad, Maryam Mehboob, and Khaled Hosseini, are among those diasporas who, through storytelling, have suggested that Afghan women are emerging, resisting, and reinventing themselves beneath the burden of marginalization and being submissive (Imran and Hart, 2018). Through storytelling, these diasporic writers bridge the gap between the realities faced by Afghan women and their stereotyped projection as well as their position in the social and cultural status of Afghanistan. Particularly, Khaled Hosseini, in his works, presented Afghan women's struggle for freedom and emancipation from patriarchal structures, despite the limits and restrictions imposed upon these women by patriarchal, social, cultural, religious, and familial structures. Therefore, his writings have also challenged the Western discourses about Afghan women and projected them as modern archetypes: obedient, resistant, and empowered. In this way, Hosseini's picture of Afghan women has reflected the fighting spirit and emancipatory attitude among the female characters of his works that reinvent them as modern archetypes, instead of traditionally subjugated, silenced, oppressed, and marginalized figures.

Khaled Hosseini, an Afghan-American novelist, was born in Kabul, Afghanistan, in 1965. Later, in 1980, his family asked for political asylum in the United States of America. By then, Afghanistan had already suffered a ruthless communist takeover and the incursion of the Soviet army, the American Invasion followed by the dreadful reign of the Taliban (Imran et al., 2020). His interest in highlighting Afghan women's quest for freedom and independent identity began during his long-awaited visit to Afghanistan in 2003. During this visit, he closely noticed the culture and society of the Afghan people and recorded some firsthand experience about the issues faced by those women during that time. Therefore, he decided to draw the attention of the whole world to the indomitable fighting spirit of the Afghan women and their desire for self-recognition and freedom.

Hosseini's (2003) debut novel, *The Kite Runner*, which marked his literary career earned the huge admiration of its readers and became a tumultuous success owing to the historical and ethnographic importance given to Afghanistan. After four years, he published his second novel, A *Thousand Splendid Suns*, in 2007, which showed the affiliation of two Afghan women with one misogynistic husband. The first two novels brought for him tremendous response from the readers; with this encouragement and motivation, he, therefore, published his third novel, *And the Mountains Echoed*, in 2013, while his fourth novel, *The Sea Prayer*, was written in 2018. His novels cover an entire gamut of problems faced by families such as the issues and themes of violence, family bonding, nationalism, male and female relationships, homeland, gender equity, and immigrants' issues, especially in Syria and Palestine (Hosseini, 2018).

The Taliban's control and the extremists' intervention in Afghan political, social, cultural, religious, and economic matters are other prominent themes highlighted by Hosseini through his novels. In his works, he exposes the affliction that Afghanistan has passed through in three decades of political unrest, conflicts, and divergence. Most of the characters in his creations show love for their homeland and express strong and passionate feelings for it. In honor of his welfare works, according to Akhtar et al. (2017), “Hosseini was appointed as goodwill envoy to UNHCR, the United Nations Refugee Agency, in 2006. Hosseini is genuinely anxious about his native country. He has been working to provide compassionate help in Afghanistan through ‘The Khaled Hosseini Foundation' which provides sanctuary to refugee families and economic and educational opportunities and healthcare for children and women from his native country” (p. 118).

In all his fiction, Khaled Hosseini attempted to voice the voiceless and support the subaltern class, particularly the women of Afghanistan. He never claims himself to be a feminist, but his writings rather showcase the ideas of a feminist approach coupled with feminist sisterhood that seeks the solidarity of women to overthrow the patriarchy and political and religious victimization of women. As feminist fiction, Hosseini's novels are a great accomplishment of a feminist cause because not only do they display the marginalization, victimization, and subjugation of women but also counteract these suppressing acts by bringing in forward bold characters that go against marginalization and fight for feminism.

The present study analyzes Hosseini's three most celebrated novels closely, *The Kite Runner, A Thousand Splendid Suns*, and *And The Mountains Echoed meticulously*, which confirm and demonstrate that he has adopted a feminist approach and consequently thereof makes conscious effort to strengthen female solidarity through the awareness of their rights, identity recognition, and feminine awakening. In Dolan's views, the feminist approach starts with a consciousness that women are excluded from male cultural, social, sexual, political, and intellectual discourse. It is a critique of the prevailing social conditions that formulate women's position as outside of the dominant male discourse (Dolan, 2012). Moreover, Lisa Gordon believes that feminism is a critique of male supremacy, formed and offered in the light of a will to change it, which in turn assumes a conviction that it is changeable (qtd. in Dolan, 2012). From a Muslim feminist, Leila Ahmed's point of view is that feminism is irreconcilably at variance with the leading philosophies in the West to more or less the same extent that it is with Islam (Ahmed, 1982). Therefore, various reactions of Muslim women are fundamentally associated with the politics of human rights and identity in Islam. According to Amrita Chhachhi, in most Muslim societies, women and their behaviors are usually taken as the signs and symbols of cultural and traditional values and markers of communal identity (Chhachhi, 1991, p. 162). In Elaine Showalter's opinion, the women's voiceless and marginalized position is the outcome of the “circumstances of being born into the wrong class, race or sex, being denied education, being numbed by economic struggle, muzzled by censorship or distracted or impeded by the demands of nurturing” (Showalter, 2002, p. 327).

The term Feminist in Afghanistan is viewed with more suspicion because of its strong leanings and connection with Western notions of sex, gender, culture, and social disruption. Being a patriarchal society where religion is manipulated to reap in personal, political, social, and traditional benefits, Afghanistan is not an easy place for the feminists, such as many women activists like Maryam Mehboob, Spojami Zaryab, and Rangina Hamidi and men like Khaled Hosseini, who agree with many feminist principles such as the protection of human rights, gender equality, women awakening and empowerment, and equal opportunities of social and economic growth for all citizens of the country. However, to announce openly as claiming to be a feminist supporter can provoke severe criticism from religious and social circles along with political marginalization (Bezhan, 2006, 2008). Therefore, for such feminist activists in Afghanistan, the most crucial undertaking is to spotlight the issues associated with women's lives, irrespective of showing their attachment with any ideology advocated by Western philosophers. In so doing, Hosseini has supported the cause of women and has given a feminist outlook and insight into his fiction. Through his experience and observation, he has aided the voice of the suppressed and subaltern in their struggle to bring about a much-needed change to support their struggle for existence socially, economically, religiously, and politically in Afghanistan.

Hosseini has encountered the continual recurrence of Afghan women's textual representation and liberal visual sensationalizing plight as third-world women who are oppressed and marginalized creatures in the Western world. Therefore, the critical works of Mani (1990), Mohanty (1991), Narayan (1997), and Spivak (1999) have endorsed Hosseini's initiative to highlight Afghan women's rebellion, emancipation, and empowerment that challenge the discursive construction of the third-world women's lives in Western accounts. Hosseini has exposed, through various bold and brave feminist narratives, the concept of traditional female slavery and seclusion domestically, as Minh-Ha (1989) describes such accounts of pain and oppression have made the women inmates in a private zoo. Hosseini and other modern Afghan writers, like Maryam Mehboob and Spojami Zaryab, have produced feminist literature that is part of the leading Afghan feminist identity writing canon that continues to address the issues of self and the politics of gender.

*A Thousand Splendid Suns* is related to the tribal and urban life's emotions and problems in Afghanistan. The novel covers the Afghans, as they swing unpredictably between happiness and disappointment, and between hope and despair while fighting with foreign and internal forces. Hosseini's poignant description of the two major female characters provides readers with a striking view of Afghanistan during the latter part of the twentieth century. Hosseini describes in detail the tyrannical regime of the Taliban, whose religiously idealistic beliefs create an unbalanced society where women are oppressed and deprived of their basic rights.

## Hosseini's women as modern archetypes

Hosseini empowers his women against patriarchal structures and makes them modern archetypes and emancipators who have the will to influence and challenge modern-day attitudes and behaviors. In Shadraconis (2013) words, “an archetype [in this sense] guides mental use of images and symbols to conform to certain themes or motifs that are found everywhere” (p.1). The following are a few examples that show how Hosseini's women behave as modern archetypes; his women believe in sanctuary and community development to establish their discourse for the fight against cruelty and injustices and independent space in society. Maryam and Laila's bond of sisterhood in *A Thousand Splendid Suns* is the best example. Hosseini's women also challenged and were emancipated from patriarchy by putting their lives at risk to achieve independence, freedom, and relief from marginalization (Ghafoor and Farooq, 2020). Nila Wahdati, Amra, and Pari in *And The Mountains Echoed* and Maryam and Laila in *A Thousand Splendid Suns* are the best examples in this regard. Hosseini portrayed these women as heroic because they did not permit the fear to make them panic and get in their way. These women bravely faced the traditional and modern techniques that patriarchal structures used to oppress, manipulate, marginalize, and subjugate. However, Hosseini's brave women preferred death, instead of life, to set an example for other community fellows. Finally, his women remain hopeful and encouraged, despite living in a male-dominated patriarchal society that opposes women's progress and independence.

## The portrayal of voiceless women in afghanistan

The position and status of women in Afghan patriarchal society are judged based on the traditional, cultural, religious, class, and political structures. These structures formulate the social roles for both men and women and define the requirements and regulations for women in a way that perpetuates patriarchal ideology. Hosseini's characters reflect the true picture of an Afghan society where the male-dominant patriarchal systems determine the gender roles; for instance, Rasheed in *A Thousand Splendid Suns* very insolently explained to Laila, his wife, that “A woman's face is her husband's business only” (Hosseini, 2007, p. 48). According to Mohanty, the fundamental complexities and conflicts characterize women's lives belonging to different cultures, classes, religions, and even societies (Mohanty, 2003, p. 20). Furthermore, in Afghan society, the positions of women are deeply affected and controlled by patriarchal ideologies in all walks of life. According to Blumberg et al. (1994), patriarchy is a theory of male authority that defines the structural and ideological methods of supremacy, in which males dominate and control females (Blumberg et al., 1994, p. 14). The patriarchal factors that support the Afghan public constitution portray women as inferior and subordinated as compared to men in all aspects of social life. Wali M. Rahimi, in his book *Status of Women: Afghanistan*, describes:

The position of women in Afghanistan has traditionally been inferior to that of men. This position has varied according to age, socio-cultural norms, and ethnicity. Afghan women, even until the beginning of the 20^th^ century, were the slaves of their father, husband, father-in-law, and elder brother. Her most valued characteristic was silence and obedience (Rahimi, 1991, p. 6).

The family structure in Afghanistan has a significant role in defining gender roles because this system gives privilege to men who have the power to determine both men's and women's status, roles, and privileges within a family. That is why boys are preferred, instead of girls, because only boys are considered to inherit the family's honor, name, and property. The image of girls is usually taken to be submissive, obedient, and dependent. Females are considered to be completely submissive to men before and after marriage, just like inanimate objects and personal property. Akhtar et al. argue that: “‘endurance' is the only way of survival in the male-oriented Afghan society. These lower-class women are constantly tortured and deprived of fully realizing their capabilities (Schrey, 2022). They lack the courage and strength to resist the standard norms of the society bluntly and are ragged between the conventional norms and their own emotions” (Akhtar et al., 2017, p. 95). According to Moghadam, in Afghan society, gender relations are deeply rooted in the concept of personal property and belonging; that is why women are considered submissive where men exercise control over them in two ways; through marriage and property and by barring landownership for women (Moghadam, 2003, p. 241).

The situations mentioned above show that the perfect image of Afghan women is only associated with their subservience and the happiness and satisfaction of the family's male member, whether he happens to be her father, elder brother, or husband. The stereotyped picture of a perfect Afghan woman is demonstrated as the one, who allows the mastery of males over her body and soul, sacrifices her complete life to serve her husband's family, and accepts the imposed male/in-laws' ideology (Joyia and Gull, 2017). Hafizullah Emadi depicts the stereotyped picture of women in Afghanistan by mentioning that the status of women is considered so inferior that men use the word “women” to insult their rivals as subservient and weak (Emadi, 1991, p. 225). The more pathetic and helpless aspect of this marginalized situation of women is that they cannot even protest against such brutalities and social injustices just because of their male dominant's honor. The concept of honor killing is also significant in this context as the male dominants are considered the symbol of family honor and so any slight disobedience to the patriarchal system brings disgrace and disrepute to their father, brother, or husband's social prestige.

Khaled Hosseini critically observed this dismal situation of women's subordination and subservience and portrayed through his major female characters the social status and various types of injustices leveled against them in his fiction. The major female characters in his novels are Jamila and Soraya in *The Kite Runner*, Mariam, Nana, and Laila in *A Thousand Splendid Suns*, and Pari, Amra, and Niala in *And The Mountains Echoed*, who demonstrate the subordination of women deeply bounded by sociocultural codes of the patriarchal system. Nevertheless, his female characters, at the climax, opposed the patriarchal system through emancipation and sought a feminist insurgence. Hosseini consciously provides his female characters with a chance to rehabilitate and challenge persistent oppression.

## Women's resistance as sanctuary

Resistance and insurgency are the primary features that Hosseini's female characters possess throughout his fiction. A rebellious attitude is, in fact, a revolt of the resisting authority, an aspect of the revolution that is a rare action to bring down the state of oppression and transform the social structure (Boswell and Dixon, 1993). Regardless of the class, status, and age, Hosseini's female characters eventually resist and rebel, and this resistance and rebellion usually happens after the oppressed class [women] have experienced class consciousness. It is a sense of awareness of belonging to a specific part of the society they are living in despite many social, cultural, religious, and traditional differences (Adamec, 1970; Hornby et al., 1995). His female characters, resisting the patriarchal system, also actively participate in the development of fellow women. These women show their resistance and rebellious attitude through various actions and expressions such as yelling, enduring, educating themselves and others, emancipating from patriarchal norms, and feminism insurgence by strengthening the sisterhood bonds and the mother–daughter relationship. Hosseini's fiction has mainly explored the general tradition and culture of gender discrimination, regardless of only the Taliban's specific regime. The significant aspect of gender discrimination in Afghan society, even before the reign of the Taliban, was extreme violence because it revealed the acts of discrimination such as rape, physical torture, and forced marriages. Such happenings imprint permanent negative and harmful impacts on the victim's social, physical, sexual, and psychological aspects of life. In Stuhr (2013) opinion, the women in Afghanistan suffered gender discrimination during several invasions to the country even before the Taliban, as Hosseini tells the stories of Afghan women stretched over decades and presents a very complex portrayal of women that goes beyond oppression and the stereotype of the veil (Stuhr, 2013, p. 53).

Hosseini's fiction explores women's resistance against many social and cultural norms along with extreme violence; sexual, psychological, and physical. In his first novel, *A Kite Runner*, he portrays the life of a typical Afghan housewife through Jamila's characters. Jamila sacrifices her whole life for the betterment of her family and does everything to make her husband happy. Before marriage, she loved to sing melodious songs, but after marriage, her husband did not allow her to sing; as Hosseini writes, “Every woman needed a husband. Even if he did silence the song in her” (Hosseini, 2003, p. 187). According to Nancy H. Dupree (1998), for a woman to disobey her parents or husband is a disgrace and dishonor because for “women, a primary obligation is to uphold family honor by confirming to accepted behavioral norms” (Dupree, 1998, p. 63). Hosseini pinpoints the cultural norms of Afghanistan that for a woman, family and husband are more important necessities than anything else. Throughout the novel, Jamila makes conscious effort to provide a better life for her daughter, Soraya, and bears all the hardships of the male-dominant society. She takes the risk of Soraya's meeting with Amir and becomes a bridge between her daughter and her husband's consensus about Soraya's marriage with Amir.

Similarly, in his second novel, *A Thousand Splendid Suns*, Mariam and Laila's characters are the best examples of women's resistance and rebellion against patriarchal ideology. At the novel's climax, Mariam decides to kill her husband Rasheed, as he is in the act of murdering Laila because Hosseini shows Mariam's insurgency that ensures Rasheed that he sees her resistance and rebellion to acknowledge her action. “He is going to kill her…He really means to. And Mariam could not… allow that to happen. He'd taken so much from her…. She wouldn't watch him take Laila too” (Hosseini, 2007, p. 301). Mariam has done this because she spent her whole married life under the constant brutality of her husband, and this is the first time she shows her resistance not only to save herself but also to save Laila's life. Hosseini, in *The Mountains Echoed*, presents a new saga of Afghan women's revolutionary actions and resistance against patriarchal society. Pari, Naila, and Amra are the featured women in this novel who bear, resist, and turn out to be intellectual and independent women. Pari's objectification in childhood and later upbringing by Naila, an educated and strong woman who shattered patriarchal influence in her teenage and strongly resisted male dominance, has changed her from a simple village girl to an educated and intelligent woman. After Naila's death, Pari emerged as Pari Wahdati and started giving lectures at the university, while Amra Ademovic dedicated herself to render volunteer work to help wartorn patients and women. Her male colleagues call her “the hardest-working woman in Kabul. You do not want to cross this girl. Also, she will drink you under the tablet” (Hosseini, 2013, p. 145). She also challenges the men who look down upon her by her wit and work as she says to her male colleague, Nabi Jan, “And I love to embarrass him” (Hosseini, 2013, p. 145). Beauvoir (2011) views that the women in the modern world accept masculine values: they pride themselves into thinking, taking action, working, and creating on the same terms as men do; instead of seeking to disgrace them, they declare themselves their equal (p. 727). Therefore, through Pari, Naila, and Amra's characters, Hosseini illustrates the radicalization of society and the revolution in modern Afghanistan.

In the stories mentioned above, the resistance and rebellion of young females take place mostly with the assistance of other females around them. For Hosseini, resistance and rebellion are not only abstract ideas but are also strongly connected with the social, cultural, religious, and political conditions of the society. However, the emancipation in women's attitudes and actions, according to Faridullah Bezhan, is attached to a prolonged war, political instability, and Islamic extremism that changed both the women's attitude from oppressed to resistant and rebellious and the Afghan society as a whole (Bezhan, 2008, p. 376).

## Terrors of war and the radicalization of afghan society

Since 1979, the Afghan society has been facing the brunt of the atrocities of Soviet attacks, the Taliban war, and local rebels, along with the US attacks after 9/11 that totally paralyzed prosperity and peace in Afghanistan. Imran and Hart (2019) have highlighted in their review essay that since the last four decades of prolonged war, foreign invaders and local militants could not succeed in getting a complete control over Afghanistan; instead, they turned the streets into battlefields, gardens into deserts, and the virtuous into causalities. Moreover, this war ravaged plight of the society exposed that the rebels and the Taliban groups did not gain public support fully because “their approach was widely deemed as an unvarying threat to the identity and well-being of the common masses and women particularly as they were their soft targets. In so doing, they have forced the women to quit their jobs, banned education, and enforced atrocious and heinous female oppression that even rebuffed the basic human rights” (Imran and Hart, 2019, p. 1). This predicament resulted in radicalization throughout the Afghan society that compelled the oppressed and subaltern class to resist and speak for their rights.

Khaled Hosseini, through an excellent craftsmanship, provides a vivid portrayal of the wars imposed by both internal and external forces and a series of ideological leaders who had traumatized the Afghan country. In his interview, Hosseini declares that he wants to write love stories and simple folk descriptions of his country, but the war and its aftershocks, along with the marginalization of the subaltern class, particularly women, compelled him to envisage the complex view of women in Afghanistan beyond the issues of oppression and the stereotype of the veil (Stuhr, 2013, p. 53). Hosseini covered the last four decades in his fiction ranging from the Pre-Soviet to the post-9/11 history of Afghanistan. In his first novel, *The Kite Runner*, the Soviet invasion and social and political unrest became the reasons for many characters to seek political asylum in neighboring and European countries. Whereas *A Thousand Splendid Suns* is also laden with details of the same period described in his first novel as the protagonist, Laila, born in April 1978, the same night when Soviet forces attacked Afghanistan and her brothers left Kabul and joined the jihadists against the Soviet army.

The whole plot of the novel revolves around war and radicalization of the society from various perspectives, including the Soviet invasion followed by local rebels seeking their control over Kabul over the Taliban's regime and, at last, the American attacks to take Kabul back from the Taliban. Hosseini describes war history and says, “the story of our country is like that one invader after another. From Macedonians to Sassanians, Arabs, and Mongols. Now there are Soviets. But our condition is like those walls up there, battered, and nothing pretty to look at, but still standing” (Hosseini, 2007, p. 132). The two female protagonists show the humiliating treatment of women after the Soviet war during the Taliban regime. Their era is characterized as harsh, tyrannical, and nightmarish for the women due to the imposed restrictions and rules. They ordered the women to wear *burka* [hide body completely under cover of cloth], and for disobedience, according to Gregory Feifer, “they were beaten for showing even an arm” (Feifer and Dean, 2009, p. 273).

During this prolonged war, political turmoil, and social unrest, Afghan society underwent rapid radicalization, and women were the first and foremost to go through this changing process. They were not only forced to oblige to put on a veil but they also had to give up their education, jobs, and even had no freedom to go to the bazaar independently without any male escort. Furthermore, Liz Kelly describes the marginalization of women in two ways; physical torture and sexual abuse. In physical torture, both men and women suffered a lot during wartime, but sexual violence damaged and tore apart women's lives completely because sexual violence is an extreme form of patriarchal control that at once constrains and damages women's lives (Kelly, 2000, p. 45). However, despite bearing all predicaments of war and patriarchy, women have developed solidarity, emancipation, and insurgency, as Hosseini has projected his female characters like Jamila, Mariam, Nana, Laila, Naila, Amra, and Pari as symbols of resistance and rebellion against tyranny to its maximum levels. The rebellious and resistant response of these women against suppression shows Hosseini's keen observation of women's situation in the context of Afghan cultural codes of conduct. However, when these women, particularly Laila, Naila, and Pari, step outside these traditional and cultural boundaries, they are groomed rapidly according to the new society's sociocultural and sociopolitical norms.

The chemistry of the relationship between Mariam and Laila and resistance and rebellion against male dominance and brutality shows the revengeful motivation and resources for extremist retaliation. During Laila's defense, Mariam's choice to kill Rasheed is considered the most important behavioral change in gender roles. In *An Essay on Liberation*, Marcuse (1969) labeled such acts as a certain degree of radical social change. Therefore, in Mariam and Laila's case, the oppression and gender exploitation have been considered an alarming rationale for social turmoil. Herbert Marcuse thinks that the triggering of such type of effervescent radical social change emerges from the reaction when someone cannot bear the oppression anymore. He further explores, “Freedom would [then] become the environment of an organism which is no longer capable of adapting to the competitive performances required for well-being under domination, no longer capable of tolerating the aggressiveness, brutality, and ugliness of the established way of life” (Marcuse, 1969, p. 9–10). The radicalization in women's attitudes is also the result of their solidarity, sisterhood, and gender-supportive attitude highlighted in almost all of Hosseini's works.

## Women's unification and confluence

Hosseini has extensively used motherhood and sisterhood bonds as primary elements for strengthening women's resistant and rebellious position, especially at every difficult time, he unites the victims to fight against oppression and brutality. Sisterhood and motherhood are widely used feminist ideas where females' connections and relations are bonded through solidarity and companionship rather than biological connection. In almost all of Hosseini's works, such sisterhood and motherhood bonds deal with the position of women and with every female, there is yet another female to support her, such as in *The Kite Runner* Jamila and Soraya's bond, in *A Thousand Splendid Suns* Nana and Mariam's bond, and in *And The Mountains Echoed* Naila and Pari's bond, which are the best-described examples of the mother [hood]—daughter relationships. While the sisterhood bond is a prevalent theme in all his novels, the most celebrated one is the bond between Mariam and Laila in *A Thousand Splendid Suns* where they help each other to make their show of resistance a sanctuary for themselves and their fellow women.

Mariam and Laila's bond of sisterhood becomes strong when they are victimized by the same person physically, psychologically, and sexually. For the first time, Laila expressed her sisterhood feeling for Mariam when Rasheed [Mariam and Laila's husband] repeatedly tortured Mariam in front of her, and she took the risk to challenge male dominance and bravely hold Rasheed's hand. Hosseini describes this situation: “Rasheed raised the belt again and this time came at Mariam. Then an astonishing thing happened: the girl lunged at him. She grabbed his arm with both hands and tried to drag him down….” (Hosseini, 2007, p. 214). Their bond went beyond the resistance, and they decided to escape together from Rasheed's oppression, but unfortunately, they remained unsuccessful as the police nabbed them in the act of escaping, and instead of helping to get rid of the spousal abuse and violence, the police again handed them over to Rasheed. He locked Laila and her daughter Aziza in an airless, suffocating hot room, while Mariam was locked away separately in a small hut. This separation infused more resistance and rebellious strength into their determination to help each other out for their survival. Later, when Rasheed repeated the physical violence against Laila that he was about to kill her that Mariam came forward and could not let him kill Laila. In Laila's defense Mariam, for the time being, forgot her ingrained servitude as a maid, cook, and housewife and emerged as Laila's savior by killing Rasheed with a sharp knife through her physical and mental strength that was aroused after many years of oppression, marginalization, sadness, and solitude (Hosseini, 2007, p. 180).

Mariam's decisive action to protect the lives of Laila and her daughter Aziza is a proof of their powerful bond of sisterhood, despite knowing the consequences. However, Mariam's role in this process of killing Rasheed is prominent as a sister to Laila and mother to Aziza, and she fully accepts the consequences of the death sentence to their lives as at the decisive moment, Laila requests her to change her mind, but she refuses and says “Think like a mother, Laila. Think like a mother. I am” (Hosseini, 2007, p. 319). According to Stuhr, Mariam's action is taken as heroic as she gives hope to others through her sacrifice and once again supports the motherhood and sisterhood narrative that she has done what mothers have always done to their family and children (Stuhr, 2013, p. 62). Through such pathetic and resistant female characters, Hosseini draws the readers' attention to domestic violence and child marriages as the primary sites of oppression for women and highlights how either parents' or in-laws' homes proved to be torturous rather than a sanctuary for them.

## Domestic violence and child marriages

The situation of women has been bleak and depressing in this inflexible Afghan society. In a society where women's only role is to produce children, they are considered worthless if they are unable to perform this childbearing duty. Women have been facing gender discrimination in its worst form for ages. Women's position was destabilized during the Soviet occupation and in the subsequent regime; in fact, the desecration of Afghan women's civil rights was considered nastiest in the early 1990s and was further battered down with the succession of the Taliban to power in 1996. Male supremacy and tyranny over women in Afghanistan are the main concerns that relate this narrative to the feminist facet.

Wollstonecraft (2014), in her work A *Vindication of the Rights of Women*, said that:

Women were human beings who should not be denied the same individual rights and privileges, including the rights to education, earnings and property ownership (p. 48).

Mary Wollstonecraft, a revolutionary feminist and women's rights advocator, attached great value to female education. She argues that through education, women can attain self-confidence and should be able to interact with the external world. She asserts further that she “sees no fundamental difference between the sexes in terms of their capacity for reason and their potential for self-development, adding that education must foster independence of thought” (Wollstonecraft, 2014, p. 48).

Throughout his fiction, Hosseini discusses domestic life and the institution of marriage as primary themes and relates these themes to the actual position of women in Afghan society. The other reason for discussing home and domestic life in Hosseini's fiction is that they play a vital role in cherishing Afghan cultural and traditional values where the majority of the women stay at home. Bezhan, in his essay, describes that even though their lives are confined to a house, they are not the master of it but only subordinate to their father, elder brother, and after marriage to their husband (Bezhan, 2008, p. 378). The cycle of violence is a part of Nana's life. Her miseries start the day her “belly began to swell” (p. 6). Jalil and his other wives cast Nana out of his home when they found out about her illicit pregnancy. Nana herself remains the victim of Jalil's humiliating behavior. Jalil never accepts her as his wife and does not give her a respectable status in society; rather he banishes her to a small hovel outside of town to live in impoverished seclusion. Rejection, abandonment, and frustrations pack her life with belligerence and depression.

Mariam also becomes the victim of such harsh treatment that had been meted out to her since childhood, which extends throughout her life until she decides to resist and put a no to the violence inflicted against her. Isolated life makes Nana an obsessed and hysteric mother. She is extremely domineering about Mariam, her only connection to the world of human beings. Nana tells Mariam, “You're all I have. I won't lose you to them…” (p. 18). Being dissipated, she has no hope of seeking and getting justice in the male-dominated Afghan society. She is harsh toward Mariam because she does not want her to face rejection from society. Nana always threatens Mariam and, in her own way, warns her about the cruelties inflicted by men and the excessive society. She presages her to learn that “a man's accusing finger always finds a woman” (p. 18).

Nana's character is placed smartly in the first part of the novel, not simply for sequential purposes but to start the novel with a feminine approach, anger, and distress. The predicament faced by Nana and Mariam is actually the story behind every woman who tries to thwart the male social order and tries to resist the system for the sake of her own identity. Amnesty International (2003) reported that women in Afghanistan are susceptible to violence in every facet of their lives, both in society and in the family unit. A female doctor elaborated in her interview with Amnesty International that familial hostility toward women is common in Afghanistan. This fact cannot be denied because most Afghan men in the region use violence. “We have a lot of cases of broken arms, broken legs and other injuries” (Amnesty International, 2003, p. 43).

However, violence against women is a social phenomenon that is deeply rooted in the dominant male society of Afghanistan. The statistics of AIHRC's [The Afghanistan Independent Human Rights Commission] reports show that domestic violence against women within the confines of the house is one of the most common forms of violence. According to the fiscal year 1396 out of 4118 cases, almost 94% of cases of violence took place in the houses against women. So, the report declares that “the home environment is the most insecure place for women in Afghanistan” (The Afghanistan Independent Human Rights Commission, 2018, p. 4). That is the reason why Hosseini symbolizes “home' as the primary site of oppression for women because, in a patriarchal society, home is a confined place where women feel imprisoned. In his first novel, *The Kite Runner*, Hosseini spotlights the reality that Jamila, in her husband's house, strove to gain basic rights. She was not even allowed to sing at home much less pursue it as a hobby because her husband did not like her singing. In the second novel, *A Thousand Splendid Suns*, first, Nana bears the spousal physical and verbal abuse, and then Mariam and Laila become the victims of Rasheed's brutality. While in *And The Mountains Echoed*, Pari's objectification in her childhood as her father sold her for a few dollars and Naila's resistance against the restrictions imposed by her father in her own home. In Afghan society, women's every act is directly associated with males' honor. Therefore, women are always more conscious about retaining the honor to be bestowed on the male heads of the family, instead of their own.

Hosseini attributes various reasons for inciting domestic violence against women, such as women's economic dependence on their fathers or husbands, making them inferior, and being treated like their property. Financial independence alone can save and even rescue women from such difficult situations, just as some empowered females in his novels prove that they can resist and challenge male dominance, such as Pari and Naila in *And The Mountains Echoed*, who after moving to Paris became financially independent and more secure in their domestic and personal lives, while Amra, by living in Kabul, gained financial independence through her job with an NGO (non-governmental organization) and enjoyed carefree self-governed life.

Ntozake Shange asserts that “every three minutes a woman is beaten, every five minutes a woman is raped, every ten minutes a little girl is molested” (qt. in Idris, p. 22); this statement clearly depicts the picture of cruelty, oppression, and violence inflicted against women. Due to their second-class status in society, women have always suffered and have been oppressed, dominated, and marginalized. Violence against women continues unabatedly to be a universal scourge that destroys, tortures, and mutilates physically, psychologically, sexually, and economically. It is a complete human rights violation to deprive women of the equality, dignity, self-esteem, and freedom they deserve throughout their lives.

Child marriage is yet another very severe and pernicious issue that is meted out to Afghan women, just as Hosseini had mentioned through Mariam's married life with Rasheed, who was of her father's age. In most of the rural areas and even in some urban areas, a majority of girls remain illiterate, and their parents arrange for their early marriages; according to Ejaz Khan qtd. in Probowati (2017), more than half of all brides are under 16 in Afghanistan, and a woman dies during giving birth to a child every half an hour. It is the country with the highest maternal death rate in the world, as up to 85 per cent of women give birth to children without any medical attention (Probowati, 2017, p. 39).

According to Rahimi and WCLRF (Women and Children Legal Research Foundation), reports in 2014 declared that the minimum age limit for girls to get married is 18 years (Rahimi, 1991; Women and children legal research foundation (WCLRF), 2014, p. 1–14), while Hosseini in *A Thousand Splendid Suns* brings to light the fact that Jalil Mariam's father arranges for her marriage when she was below the age of 15 years, while her husband's age in contrast was 45 years. The other incident portraying a clear defiance of age rule can be spotted out in Laila and Raheed's marriage, where their ages were 14 and 63 years, respectively. Such young or early marriages bring in many complexities to premature young wives. Therefore, most of the time, the young wives are totally dependent on their husbands and patiently bear their dominance and oppression. Consequently, their oppression infuses in them a strong urge for emancipation from patriarchal control, and their feminist insurgence awakes inside them a strong resistant and rebellious vim.

## Conclusion

The modern English fiction on Afghanistan presents the factual picture of Afghanistan based on the framework of patriarchal systems and gender disparity. The characters analyzed in this article explore, despite the reality, that the new generation, particularly of women, wants a change in social, cultural, and political behaviors. The obedient and resistant Afghan women have shown through their emancipation and insurgency that unless the elimination of gender disparity and marginalization along with the promotion of gender solidarity and friendliness takes place, the betterment and development of third-world men and women remain a distant dream. Hence, educated and well-informed writers like Khaled Hosseini have advocated for and heralded in forging the emerging and new mindset among youth and the subaltern class to resist and demand their basic rights.

Therefore, Hosseini has attempted to voice the voiceless and show courage to the discouraged women and has promoted an atmosphere of companionship and gender-friendliness to sympathize with each other. Through his fictional characters, Hosseini presents a unique change in the attitudes of Afghan women, very different from the stereotyped images presented in global media where Afghan women are shown to be only victimized, oppressed, abducted, and subordinated. Hosseini's females represent the life of ordinary women who are subjected to and suffer from various forms of violence, oppression, and subservience, and at last break the manmade skeleton of sociocultural and sociopolitical hegemonic control over their bodies and souls. Hosseini has equipped and endowed his women with a rebellious and resistant nature that not only helps them achieve relief from tyranny and marginalization but also gives them the courage and fortitude to struggle for others.

## Data availability statement

The original contributions presented in the study are included in the article/supplementary material, further inquiries can be directed to the corresponding author.

## Author contributions

Both authors have equally participated in the completion of this work and have approved it for publication.

## Conflict of interest

The authors declare that the research was conducted in the absence of any commercial or financial relationships that could be construed as a potential conflict of interest.

## Publisher's note

All claims expressed in this article are solely those of the authors and do not necessarily represent those of their affiliated organizations, or those of the publisher, the editors and the reviewers. Any product that may be evaluated in this article, or claim that may be made by its manufacturer, is not guaranteed or endorsed by the publisher.

## Footnotes

^1^Burqa: a traditional long dress that is used to cover the whole body of the female, except some parts of eyes in Afghanistan.
